# Supplementary material for: Employment status and its associated factors for patients 12 months after intensive care: Secondary analysis of the SMAP-HoPe study
Source: PLoS One. 2022 Mar 18;17(3):e0263441. doi: 10.1371/journal.pone.0263441 (PMC8932587; doi:10.1371/journal.pone.0263441)
Supplement: S1 Table — (DOCX) [file pone.0263441.s001.docx]

**S1　Table. Characteristics and number of analyzed participants for each intensive care unit**

|  | **Center** | | | | | | | | | | | |
| --- | --- | --- | --- | --- | --- | --- | --- | --- | --- | --- | --- | --- |
| **Hospital type** | Private | University | University | Private | University | Public | University | University | Public | University | University | University |
| **Name and location of participant institution** | Japanese Red Cross Fukuoka Hospital, Fukuoka, Fukuoka | Jichi Medical University Hospital, Shimotsuke, Tochigi | Kanazawa Medical University Hospital, Kahoku, Ishikawa | Sakakibara Heart Institute, Fuchu, Tokyo | Japanese Red Cross Maebashi Hospital, Maebahi, Gunma | Nara Medical University Hospital, Kashihara, Nara | Nara Medical University Hospital, Kashihara, Nara | Nippon Medical School Musashikosugi Hospital, Kawasaki, Kanagawa | Osaka City General Hospital, Osaka, Osaka | Sapporo Medical University Hospital, Sapporo, Hokkaido | Tohoku Medical and Pharmaceutical University Hospital, Sendai, Miyagi | University of Tsukuba Hospital, Tsukuba, Ibaraki |
| **ICU type** | med-surg | med-surg | med-surg | CV surgery | med-surg and emergency | med-surg | med-surg | med-surg | med-surg | med-surg | med-surg | med-surg |
| **No. of ICU beds** | 8 | 16 | 8 | 12 | 16 | 8 | 14 | 6 | 16 | 6 | 10 | 12 |
| **Participants, n (%)** | 25 (7.6) | 28 (8.5) | 21 (6.4) | 31 (9.5) | 26 (7.9) | 17 (5.2) | 35 (10.7) | 42 (12.8) | 46 (14.0) | 1 (0.3) | 22 (6.7) | 34 (10.4) |
| **Duration for enrollment** | 10 months | 7 months | 9 months | 10 months | 9 months | 9 months | 10 months | 10 months | 9 months | 10 months | 10 months | 9 months |

CV surgery, cardiovascular surgery; Med-Surg, medical-surgical; ICU, intensive care unit
