# Supplementary material for: Employment status and its associated factors for patients 12 months after intensive care: Secondary analysis of the SMAP-HoPe study
Source: PLoS One. 2022 Mar 18;17(3):e0263441. doi: 10.1371/journal.pone.0263441 (PMC8932587; doi:10.1371/journal.pone.0263441)
Supplement: S3 Table — (DOCX) [file pone.0263441.s003.docx]

**S3 Table. Sensitivity analysis**

Multivariable analysis of factors associated with unemployed status 12 months after intensive care unit discharge among patients after excluding patients aged 60 years or younger

| **Variable** | **Odds Ratio** | **95% CI** | **p** |
| --- | --- | --- | --- |
| **Age** | 1.04 | 0.99-1.10 | 0.129 |
| **Male** | 0.93 | 0.31-2.76 | 0.898 |
| **Previous employment status** | | | |
| **Part-time employed** | 1.29 | 0.57-2.93 | <0.537 |
| **Self-employed** | 0.17 | 0.07-0.41 | <0.001 |
| **Cognitive impairment** | 0.79 | 0.20-3.08 | 0.731 |
| **Physical dysfunction** | 1.90 | 0.57-6.26 | 0.294 |
| **Severity of depression** | 1.17 | 1.06-1.30 | 0.002 |

CI, Confidence Interval
