# Supplementary material for: Employment status and its associated factors for patients 12 months after intensive care: Secondary analysis of the SMAP-HoPe study
Source: PLoS One. 2022 Mar 18;17(3):e0263441. doi: 10.1371/journal.pone.0263441 (PMC8932587; doi:10.1371/journal.pone.0263441)
Supplement: S1 Text — (DOCX) [file pone.0263441.s004.docx]

**S1 Text**

**Questionnaires regarding employment status, house finance, and subjective cognitive function**

病院入院前と現在で、仕事や経済状態などの生活、認知機能に変化があったかをお伺いします。もっともよくあてはまる選択肢の□にチェック︎をつけてください。

①病院に入院する前の就業状況に関して教えてください。

□無職

□自営業

□会社員等、正規職員

□非正規職員

□その他

①現在の就業状況に関して教えてください。

□無職

□自営業

□会社員等、正規職員

□非正規職員

□その他

③入院前と比べて、家庭の経済状況は悪化しましたか？

□悪化した

□変わらない

□良くなった

④入院前と比較して、ものごとに集中しにくいことがありますか？例えば新聞を読むときや、テレビを見るときなど。

□とても多い

□多い

□少しある

□ない

⑤入院前と比較して、もの覚えが悪くなったと思うことがありますか。

□とても多い

□多い

□少しある

□ない

-------------------------------------------------------------------------------

**English-version**

We would like to ask you if there have been any changes in your life and cognitive functioning, such as your job and financial status, between before and after your hospitalization. Please check the box that best describes your situation.

(1) Please tell us about your employment status before you were admitted to the hospital.

□Unemployed

□Self-employed

□Full-time employee

□Part-time employee

□Other

Please tell us about your current employment status.

□Unemployed

□Self-employed

□Full-time employee

□Part-time employee

□Other

(3) Has your house financial situation worsened compared to before your hospitalization?

□Worse

□No change

□Better

(4) Compared to before your hospitalization, how often do you think your memory function was impaired compared with before hospital admission? For example, when you read the newspaper or watch TV.

□Very frequently

□Frequently

□Sometimes

□Not at all

(5) How often do you feel your concentration function was impaired compared with before hospital admission? For example, how do you feel when you are reading a newspaper or watching TV?

□Very frequently

□Frequently

□Sometimes

□Not at all
